# The effect of acorn muffin consumption on glycemic indices and lipid profile in type 2 diabetic patients: A randomized double‐blind placebo‐controlled clinical trial

**DOI:** 10.1002/fsn3.3123

**Published:** 2022-11-15

**Authors:** Najmeh Sasani, Asma Kazemi, Siavash Babajafari, Ehsan Amiri‐Ardekani, Mojtaba Rezaiyan, Reza Barati‐Boldaji, Seyed Mohammad Mazloomi, Cain C. T. Clark, Elham Ashrafi‐Dehkordi

**Affiliations:** ^1^ Nutrition Research Center, School of Nutrition and Food Sciences Shiraz University of Medical Sciences Shiraz Iran; ^2^ Nutrition Research Center Shiraz University of Medical Sciences Shiraz Iran; ^3^ Department of Phytopharmaceuticals (Traditional Pharmacy) Shiraz University of Medical Sciences Shiraz Iran; ^4^ Centre for Intelligent Healthcare Coventry University Coventry UK

**Keywords:** acorn, appetite, diabetes, functional food, oak, Quercus

## Abstract

Acorn is a nutritious fruit with the reported potential of ameliorating diseases, including diabetes. This research aimed to assess the effects of acorn muffin consumption on glycemic, lipid indices, and appetite in patients with type 2 diabetes. Sixty‐six subjects were dichotomized to receive either one muffin containing 10 grams of acorn flour or a placebo muffin containing white wheat flour (no bran), per day, for 8 weeks. Acorn muffin consumption improved glycated hemoglobin (*p* = .06, mean difference [MD] = −0.65), triglyceride (*p* = .06, MD = −36.38), and high‐density lipoprotein (*p* = .05, MD = 1.30), albeit only marginally significantly. Among appetite parameters, hunger, desire to eat, and prospective to eat were significantly lower, and satiety and fullness were significantly higher, in the acorn muffin group. In conclusion, acorn muffins could be utilized as an adjuvant therapy to control appetite and ameliorate glycated hemoglobin in patients with type 2 diabetes. However, further investigations are required for a more comprehensive conclusion.

## Graphical abstract

Acorn muffins could be utilized as an adjuvant therapy to control appetite and ameliorate glycated haemoglobin in patients with type 2 diabetes. However, further investigations are required for a more comprehensive conclusion.

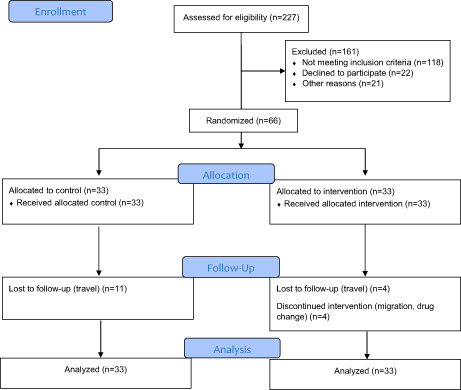

## 1 INTRODUCTION

Despite the presence of various approaches for controlling diabetes mellitus (DM), such as dietary adjustment (Asif, 2014) and hypoglycemic agents, some patients do not receive the full advantages of current methods due to several restrictions, such as medication costs, accessibility, and concern about the adverse effects of drugs (Sasani et al., 2021). Due to the potentially severe complications of DM, recognizing new applicable approaches for prevention and controlling DM is critical. Identifying and utilizing functional foods (FFs) is one of these new strategies (Mirmiran et al., 2014), where FFs are products in the form of food, not drugs, which provide positive health effects when they are consumed in a normal amount in a usual diet (Amiri Ardekani et al., 2020).

Throughout history, acorn, the oak fruit, has been considered as a food and is a part of the usual daily diet in various regions, globally (Burlacu et al., 2020). Acorn contains notable amounts of carbohydrates, protein, and unsaturated fatty acids, such as oleic acid (Taib & Bouyazza, 2021). Moreover, it contains a high amount of phenolic compounds, saponins, and tannins (Şöhretoğlu & Sakar, 2004), which confers antidiabetic effects by enhancing glycolytic enzymes' activity (Burlacu et al., 2020) and could regulate lipid profile (Taib et al., 2020). As previously noted, acorn is a good source of oleic acid, which has been demonstrated to have beneficial effect on DM and blood lipid level (Taib et al., 2020). Moreover, in‐vivo and in‐vitro investigations have shown hypoglycemic properties of acorn extract, such as α‐amylase inhibition, and positive effects of acorn on DM complications (Dogan et al., 2015; Shaheen et al., 2017).

Recently, adding acorn into the human diet has yielded scientific interest (Pereira & Oliveira, 2018). Thus, in the current study, we examined the effects of acorn muffin consumption on glycemic indices, lipid profile, oxidative stress, and anthropometric indices in patients with type 2 diabetes.

## 2 MATERIALS AND METHODS

### 2.1 Materials

Acorn *(Q. brantii* Lindl), identified by S. Khademian, was obtained from the forest of Fars province in southern Iran. The associated voucher specimen (3041) is kept in the herbarium of Pharmacy school at Shiraz University of Medical Sciences. White wheat flour (WF) (no bran) was purchased from the Tarkhineh Co. in Iran. Egg, stevia, low‐fat milk, canola oil, baking powder, emulsifier, brown food color, and vanilla essence were acquired from local supermarkets.

### 2.2 Acorn flour preparation

The shell and internal layer of acorns were removed and thereupon the fruits were soaked in water for 48 h to reduce the astringent taste. Finally, the acorns were dried in an ambient condition for 30 days and ground by a hammer mill.

### 2.3 Muffin's preparation

Eggs and sugar were whipped together with a kitchen‐aid mixer at low speed. Then, vanilla essence, canola oil, low‐fat milk, emulsifier, flour, and baking powder were added, respectively, and all the ingredients were mixed together. Muffin batter was poured into a paper baking cup and backed at 180°C for 30 min. Finally, muffins were cooled at ambient temperature. All ingredients were similar and equal in amount, for both intervention and control muffins, except flour. Ten grams of acorn flour was used in the preparation of each intervention muffin. WF was used in the control muffins instead of acorn flour. Given that the calorie content of acorn flour was more than WF, we applied about 12 g WF to make the calories of both muffins equal. Each muffin weighed about 30–35 g. Because of the natural brown color of acorn muffins, brown food color was used to make the muffins visually similar.

### 2.4 Chemical analyses of flour and muffins

AACC (2000) methods were applied to determine protein, fat, moisture, ash, and fiber contents. By subtracting the sum of fat, protein, ash, and moisture from 100%, the total carbohydrates quantity was obtained. Tannins of acorn muffin were measured by titration method and application of indigo solution as an identifier (1). The energy values were calculated based on Atwater coefficients (carbohydrates and protein 4 kcal/g, fat 9 kcal/g) (Maclean et al., 2003). All analyses were performed two times.

### 2.5 Ethical issues

This study was conducted according to the guidelines of the Declaration of Helsinki and all procedures involving human patients were approved by the Ethics Committee of Shiraz University of Medical Sciences, Shiraz, Iran (with reference number: IR.SUMS.REC.1398.1005). Written informed consent was obtained from all patients. This trial was registered in the Iranian Registry of Clinical Trials (registration reference: IRCT20191009045042N1).

### 2.6 Trial design, randomization, and blinding

The study was designed as a randomized double‐blinded (participants and investigator) controlled‐clinical trial with a duration of 8 weeks. Participants were stratified based on gender, age, and glycated hemoglobin (HbA1c) (HbA1c ≤ 7, HbA1c >7) and allocated the two groups by block randomization with a block size of two. A person, who had no role throughout the study, labeled the intervention and control muffins packages as “A” and “B”.

### 2.7 Inclusion and exclusion criteria

Patients with type 2 diabetes (male and female), aged between 30 and 65 years, were included in the study. Other eligibility criteria were fasting blood glucose (FBG) ≤ 250 mg/dl, only taking oral hypoglycemic agents, not having constipation, not taking a multivitamin, mineral, and/or antioxidant supplements over the 3 months preceding the study, were not on a specific diet (vegetarian, etc.), were not pregnant or lactating, not drinking alcohol, and agreed not to change physical activity and dietary patterns throughout the study period. Participants were excluded if their medication changed during the study, and if they did not consume more than 20% of the muffins.

### 2.8 Intervention

Subjects who had clinical data in the Shiraz “Ghotbeddin diabetes clinic” were contacted by telephone in January 2020. Research goals and investigation methods were explained to them. After the selection of patients, they were invited to the clinic lab, and demographic, clinical characteristics, and other questionnaire‐based information were collected. Patients were requested to eat a muffin once a day for 2 months. Participants were reminded to consume the muffin daily by sending a WhatsApp message. A follow‐up phone call was made every 2 weeks to (re)emphasize eating the muffins and checking for any potential side effects, and lifestyle or medication changes. A checklist was provided to the participants, and they were asked to record a checkmark after eating each muffin. After two months, patients were visited again and the indicators measured at the beginning of the study were re‐evaluated. No change was made in the routine medication/treatment regimens of participants during the study.

### 2.9 Outcome measurements

Anthropometric indices, including weight, height, body mass index (BMI) (kg/m^2^), and waist circumference, were measured before and after the study. Weight was measured by using a digital scale (Seca, Hamburg, Germany) (0.5 kg accuracy), with participants unshod and in light clothing. Height was measured using a stadiometer, to the nearest 1 centimeter. Waist circumference was obtained by measuring the length of the circumference of the smallest area below the chest and above the navel using a nonelastic measuring tape (0.5 cm accuracy).

Physical activity was evaluated using the short version of the international physical activity questionnaire before and after the study.

Appetite was assessed, while participants were in a fasted state, using a 10‐cm visual analog scale, at baseline and end of the intervention. Participants were asked to rate, on the scale, their feeling at that particular time about hunger, satiety, fullness, desire, and prospective to eat from 0 (not at all) to 10 (extremely). The intensity of the feeling (the distance of the point from the origin on the right) was measured, yielding a score from 0 to 10.

Dietary intake was registered by a dietitian at the beginning and end of the study (one weekend and two working days) using a 24 h food recall questionnaire. The average of three food recalls was converted to grams and entered into Nutritionist IV software (based on the food composition table of Agriculture Department of US, modified for Iranian foods).

A blood sample was drawn after 12 h fasting at the beginning and end of the study. FBG was measured by an enzymatic colorimetric (GOD‐PAP) methodology (Pars Azmoon Inc, Iran). HbA1c was assessed by immunoturbidimetric assay (Beckman Coulter Synchron LX®20, Brea, CA, USA). Serum insulin level was measured by Enzyme‐linked Immunosorbent Assay (ELISA) (monobind, US). The subsequent equation used to calculate insulin resistance based on the homeostasis model of insulin resistance index (HOMA‐IR): [FBG (mg/dl) * Serum insulin level (mIU/L)]/405 (Matthews et al., 1985). 2 h after consuming a usual breakfast, another blood sample was drawn to assess 2 h postprandial (2hpp) glucose. Triglycerides (TG), total cholesterol (TC), and high‐density lipoprotein (HDL) were analyzed via an automatic analyzer using specific assay kits (Pars Azmun, Iran). Low‐density lipoprotein (LDL) was evaluated by FriedeWald's formula. Serum malondialdehyde (MDA) was measured via the modified thiobarbituric acid method (spectrophotometric), and serum total antioxidant capacity (TAC) was determined by colorimetric assay (Biocore diagnostics, Ham‐burg, Germany).

### 2.10 Sample size calculation and statistical analysis

The sample size was determined to be 27 patients in each group on the basis of the following assumptions: a mean difference of 0.5 (equivalent to minimum clinically important difference [MICD]) (Goldenberg et al., 2021), study power of 80%, and standard deviation of 0.7 and 0.75. Moreover, by considering a potential 20% dropout in subjects, 66 patients were enrolled in the study.

STATA software version 14 (StataCorp LP, College Station, TX, USA) was used to analyze data. Variables were reported based on mean ± standard deviation or number (percentage) where necessary. A Shapiro–Wilk test was used to assess the normality distribution of data, and, where necessary, logarithmic transformation was applied to fit parameters in a normally distributed model. A Chi‐square test was used to determine the difference between groups at baseline for categorical data, and analysis of covariance (ANCOVA), with adjustment for covariates (baseline values of each outcome and calorie intake), was performed with the intention‐to‐treat (ITT) approach, between groups, at the end of the study. *p* values <0.05 were considered statistically significant.

## 3 RESULTS

### 3.1 Chemical analysis

Based on the Tarkhineh Co. reports, 100 g of WF has 350 kcal energy and the flour contains no fiber. Table 1 depicts the chemical analyses of muffins and acorn flour.

**TABLE 1:** Nutritional composition of muffins and acorn flour per 100 g

Sample	Protein (W.P.)	Pro NRV (W.P.)	Fat (W.P.)	Fiber (%)	Ash (W.P.)	Moisture (W.P.)	Total CHO (%)	Tannins (g)	Energy (kcal)
Acorn cake	5.575 *	11.15	16.81	0.505	1.63	44.25	31.22	0.054	303.38
Control cake	7.26	14.53	15.02	Not detected	1.32	44.89	31.49	Not detected	295.63
Acorn flour	4.9	9.8	6.2	1.76	1.165	6.74	79.23	Not examined	399.9

Abbreviations: CHO, Carbohydrate; Pro NRV, Protein nutrient reference value; W.P., Weight percent.

^a^ Data reports as mean.

### 3.2 Study flow

**FIGURE 1:**
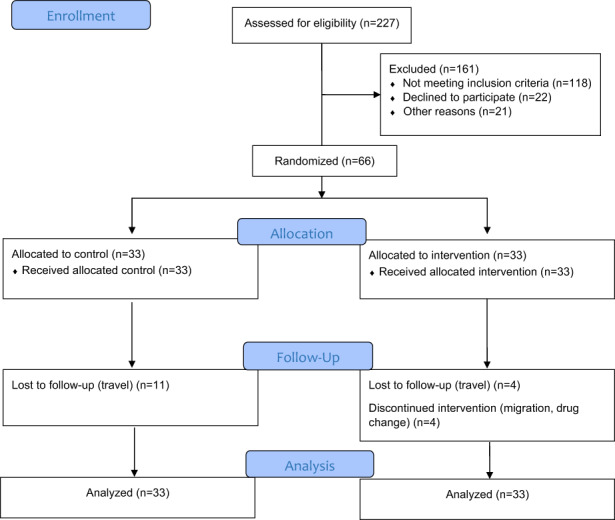
The CONSORT flow diagram

A total of 66 eligible patients were randomized into the intervention and control groups from January 2020 to March 2020. As shown in Figure 1, during the study, four participants withdrew from the study. Also, at the end of the study, we could not get access to 15 patients because they had traveled to their homeland with the onset of the COVID‐19 pandemic. Therefore, 47 participants completed the study. Based on post‐hoc power estimation for the FBG, 2hpp glucose, and HbA1c, the power of the study (1‐beta coefficient) was between 0.7 and 1.

Baseline demographic and clinical characteristics of patients are presented in Table 2, and there was no significant difference between groups in any characteristics at baseline.

**TABLE 2:** Baseline demographic and clinical characteristics

Characteristics	Intervention group (*n* = 33) mean ± SD	Control group (*n* = 33) mean ± SD	*p*
Age (years)	53.93 ± 7.54	57.12 ± 7.12	.08*
Gender†			0.42^a^
Female	24 (72.73%)	21 (63.64%)	
Male	9 (27.27%)	12 (36.36%)	
Duration of diabetes (year)	6.87 ± 5.29	9.06 ± 7.13	0.16^b^
Weight (kg)	74.46 ± 15.97	73.73 ± 17.44	0.86^b*^
WC (cm)	93.89 ± 13.76	96.21 ± 12.17	0.47^b*^
BMI (kg/m^2^)	29.25 ± 5.35	29.35 ± 5.39	0.93^b*^
Physical activity (MET.min/week)	1253.15 ± 1345.12	1147.97 ± 2194.70	0.81^b*^
Menopause†	19 (82.61%)	17 (80.95%)	0.88^a**^

Abbreviations: BMI, body mass index; MET, metabolic equivalent; WC, waist circumference.

^a^ Chi‐Square test.

^b^ Independent t‐test.

^c^ Number (%).

### 3.3 Glycemic indices

The change in HbA1c was marginally significant between the two groups (MD: −0.65%, 95% CI: −1.40 to 0.09, *p* = .065). FBG (MD: −21.78 mg/dl, 95% CI: −44.84 to 1.28, *p* = .09), 2hpp (MD: −34.11 mg/dl, 95% CI: −63.56 to −4.67, *p* = 0.10), insulin (MD: −4.36 mIU/L, 95% CI: −11.25 to 2.51, *p* = 0.63), and HOMA‐IR (MD: ‐2.19, 95% CI: −5.60 to 1.21, *p* = 0.50) levels did not change significantly between the two groups (Table 3).

**TABLE 3:** Glycemic indices before and after the study

Variable	Intervention group (*n* = 33) mean ± SD	Control group (*n* = 33) mean ± SD	*p* ^a^
FBG† (mg/dl)			.09
Before	144.27 ± 49.78	139.27 ± 48.54	
after	126.69 ± 38.21	143.48 ± 37.59	
2hpp† (mg/dl)			0.10
Before	207.78 ± 72.54	213.63 ± 89.75	
after	184.59 ± 47.40	224.56 ± 66.13	
HbA1c† (%)			0.06
Before	7.1 ± 1.53	6.95 ± 1.49	
After	6.20 ± 1.14	6.71 ± 1.35	
Insulin† (mIU/L)			0.63
Before	12.03 ± 10.98	10.19 ± 6.85	
After	12.04 ± 7.95	14.57 ± 15.99	
HOMA‐IR†			0.50
Before	4.35 ± 5.30	3.71 ± 3.10	
After	3.90 ± 3.91	5.46 ± 7.65	

Abbreviations: FBG, fasting blood glucose; 2hpp, 2 h postprandial; HbA1c, glycated hemoglobin; HOMA‐IR, homeostatic model assessment of insulin resistance.

^a^ ANCOVA (adjusted for calorie intake).

^b^ Log transformed.

### 3.4 Lipid profile

As shown in Table 4, the change in TG (MD: −36.38 mg/dl, 95% CI: −65.46 to −7.30, *p* = .062) and HDL (MD: 1.30 mg/dl, 95% CI: −1.61 to 4.23, *p* = .055) levels was marginally significant between the two groups. TC (MD: −33.74 mg/dl, 95% CI: −49.23 to −18.25, *p* = 0.25) and LDL (MD: −28.13 mg/dl, 95% CI: −41.84 to −14.42, *p* = 0.52) levels did not change significantly between the two groups.

**TABLE 4:** Lipid profile and oxidative stress before and after the study

Variable	Intervention group (*n* = 33) mean ± SD	Control group (*n* = 33) mean ± SD	*p* *
TG† (mg/dl)			.06
before	157.87 ± 84.57	135.78 ± 53.66	
after	148.70 ± 45.16	162.99 ± 42.64	
TC† (mg/dl)			0.25
before	177.90 ± 34.67	156.06 ± 41.75	
after	164.93 ± 20.48	176.83 ± 40.57	
LDL† (mg/dl)			0.52
before	98.45 ± 32.16	82.63 ± 34.84	
after	86.35 ± 20.89	98.67 ± 38.61	
HDL† (mg/dl)			0.05
before	48.66 ± 7.18	47.27 ± 6.96	
after	48.99 ± 4.55	46.28 ± 4.86	
MDA (μM/L)			0.89
before	2.09 ± 0.69	2.06 ± 0.68	
after	1.88 ± 0.70	2.09 ± 0.63	
TAC (μM/L)			0.54
before	1.81 ± 0.35	1.84 ± 0.31	
after	1.76 ± 0.27	1.77 ± 0.35	

Abbreviations: HDL, High‐density lipoprotein; LDL, Low‐density lipoprotein; MDA, malondialdehyde; 'TAC, total antioxidant capacity; TC, total cholesterol; TG, triglyceride.

^a^ Log transformed.

^b^ ANCOVA (adjusted for calorie intake).

### 3.5 Antioxidant markers

The analyses showed no statistically significant changes in TAC (MD: 0.01 μM/L, 95% CI: −0.21 to 0.23, *p* = 0.54) and MDA (MD: −0.23 μM/L, 95% CI: −0.75 to 0.27, *p* = 0.89) between the two groups (Table 4).

### 3.6 Anthropometric measurement

No significant change was observed in BMI (mean difference (MD): 1.06 kg/m^2^, 95% confidence (CI): −0.30 to 2.42, *p* = 0.45), weight (MD: 1.06 kg, 95% CI: −0.30 to 2.42, *p* = 0.77), waist circumference (MD: −0.68 cm, 95% CI: −1.09 to −0.27, *p* = 0.15), between the two groups. (Table 5).

**TABLE 5:** Anthropometric indices and physical activity before and after the study

Variable	Intervention group (*n* = 33) mean ± SD	Control group (*n* = 33) mean ± SD	*p*
BMI (kg/m^2^)			0.45*
before	29.25 ± 5.35	29.35 ± 5.39	
after	29.76 ± 4.75	28.80 ± 5.19	
Weight† (kg)			0.77*
before	74.46 ± 15.97	73.73 ± 17.44	
after	74.07 ± 15.43	73.96 ± 17.56	
WC† (cm)			0.15*
before	93.89 ± 13.76	96.21 ± 12.17	
after	93.29 ± 13.68	96.30 ± 12.34	
Physical activity† (MET.min/week)			0.45*
before	1253.15 ± 1345.12	1147.97 ± 2194.70	
after	1415.42 ± 1758.48	1404.40 ± 2585.84	

Abbreviations: MET, metabolic equivalent.

^a^ Log transformed.

^b^ ANCOVA (adjusted for calorie intake).

### 3.7 Appetite parameters and dietary intake

Hunger (MD: −0.75 cm, 95% CI: −1.79 to 0.28, *p* = .04), desire to eat (MD: −2.18 cm, 95% CI: −3.37 to −0.99, *p* = .000), and prospective to eat (MD: −2.58 cm, 95% CI: −3.49 to −1.67 *p* = .001) were significantly lower in the acorn muffin group. While satiety (MD: 1.18 cm, 95% CI: 0.28 to 2.08, *p* = .003) and fullness (MD: 1.24 cm, 95% CI: 0.32 to 2.15, *p* = .003) were significantly higher in the acorn muffin group.

The changes in calorie intake, carbohydrate intake, and fat intake were significantly different between the two groups (*p* = .000, 0.03, and 0.02, respectively) (Table 6). Further, participants reported no adverse effects throughout the intervention.

**TABLE 6:** Appetite indices and dietary intake before and after the study

Variable	Intervention group (*n* = 33) mean ± SD	Control group (*n* = 33) mean ± SD	*p* *
Hunger (cm)			.04
before	4.24 ± 2.89	4.09 ± 3.39	
after	3.69 ± 2.86	4.30 ± 2.94	
Fullness† (cm)			.003
before	3.12 ± 2.59	2.24 ± 3.00	
after	3.96 ± 2.17	1.84 ± 2.56	
Satiety (cm)			.003
before	4.45 ± 2.70	4.21 ± 3.23	
after	5.66 ± 2.61	4.24 ± 2.57	
Desire to eat(cm)			.000
before	5.48 ± 2.46	4.72 ± 3.05	
after	3.90 ± 2.21	5.33 ± 2.74	
Prospective to eat (cm)			.001
before	6.33 ± 2.30	5.60 ± 3.02	
after	4.96 ± 2.11	6.31 ± 2.58	
Calorie intake^†^ (kcal)			.000**
before	2261.57 ± 218.79	2208.66 ± 322.31	
after	2138.36 ± 202.76	2226 ± 366.09	
CHO intake† (gr)			.03**
before	307.63 ± 60.67	308.79 ± 49.63	
after	292.872 ± 31.23	312.46 ± 50.74	
Protein intake† (gr)			.32**
before	73.88 ± 14.98	67.97 ± 15.28	
after	76.83 ± 8.25	74.06 ± 12.02	
Fat intake† (gr)			.02**
before	82.01 ± 10.55	81.02 ± 15.62	
after	77.23 ± 9.08	79.21 ± 17.29	
Fiber intake† (gr)			.62 **
before	20.15 ± 6.53	19.46 ± 4.71	
after	20.11 ± 5.45	19.15 ± 3.64	

^a^ Log transformed.

^b^ ANCOVA (adjusted for calorie intake).

^c^ ANCOVA.

## 4 DISCUSSION

Daily consumption of acorn flour muffins, containing 10 g acorn flour, improved HbA1c, HDL, and TG marginally significantly, and also led to significantly **greater satiety levels.**

### 4.1 Effects of acorn muffin on glycemic indices

In the current trial, a marginally significant effect was seen in HbA1c. Although other parameters of glycemic indices did not change significantly, in comparison with prespecified MICD (Goldenberg et al., 2021), the change in HbA1c, insulin, and HOMA‐IR were clinically important. Based on this result, it is suggestible that acorn muffin could yield some beneficial effects on glycemic control. Indeed, it should be noted that the duration of our study was only 8 weeks, and if the period were longer, perhaps additional significant effects would have been seen.

Nevertheless, the beneficial effect found in this study could be related to the effects of acorn flour tannins that can influence carbohydrate digestion and also increase fullness, thereby ameliorating postprandial glucose.

Dogan et al. investigated the hypoglycemic effects of *Q.brantii* acorn extract in diabetic rats (Dogan et al., 2015), and the results showed an improvement in serum glucose, serum insulin, and HbA1c. Further, the greater the amount of Quercus extract level, the better the observed glycemic improvement (Dogan et al., 2015). Also, Shaheen et al. (Shaheen et al., 2017) reported the positive effects of the *Q. dilatata* extract on blood glucose levels of diabetic rats. However, contrary to the study of Dogan et al., in Shaheen et al., the hypoglycemic effects were more noticeable in the rats treated with 200 mg/kg extract than the rats treated with 400 mg/kg of extract. Furthermore, in the assessment of Quercus extract effect in normoglycemic rats, Shaheen et al. showed a statistically significant reduction in blood glucose, highlighting the potential hypoglycemic effects of Quercus extract. In another study, where the effects of *Q. ilex* acorn aqueous extract were assessed, a dose‐dependent reduction in glucose‐induced short circuit current was highlighted. Moreover, these results were authenticated by assessing the effects of acute oral administration of *Q.ilex* aqueous extract after taking 2 g/kg glucose in healthy rats, which demonstrated a dose‐dependent reduction in glucose concentration of rats' blood (Rtibi et al., 2017).

### 4.2 Possible mechanism of acorn's hypoglycemic effect

Acorn is a rich source of components possessing hypoglycemic activity, including saponins and phenolic compounds, such as flavonoids and tannins (Şöhretoğlu & Sakar, 2004). According to the available evidence, tannins can inhibit α‐amylase and α‐glucosidase activity (Barrett et al., 2013; Xiao et al., 2015), thereby reducing the rate of carbohydrate absorption and consequently reducing glucose in the bloodstream and leading to a better postprandial glucose control (Ajebli & Eddouks, 2019). Tannic acid also reduces the absorption of glucose by affecting SGLUT1 in the small intestine (Hanhineva et al., 2010); indeed, Dogan et al. suggested that Quercus can improve glucose metabolism by increasing glycolysis and reducing gluconeogenesis (Dogan et al., 2015). As Quercus is a good source of nondigestible fiber, one of the mechanisms of hypoglycemia may be related to its presence. Fiber improves postprandial glucose by delaying digestion and absorption of carbohydrates (Post et al., 2012), and also can increase peripheral insulin sensitivity by fermenting in the small intestine and producing short‐chain fatty acids (Johnston et al., 2009; Robertson et al., 2012).

### 4.3 Effects of acorn muffin on lipid profile

In the current study, a marginally significant effect was seen in TG and HDL between groups. In comparison with prespecified MICD (Goldenberg et al., 2021), the changes in TG, TC, and LDL were clinically important. Studies of Dogan et al. (Dogan et al., 2015) and Shaheen et al. (Shaheen et al., 2017) reveal an improvement in levels of HDL, LDL, TC, and TG, where the higher Quercus extract doses yielded better improvements. The abovementioned studies suggested that the mechanism could be associated with the decrement in cholesterogenesis and synthesis of fatty acid, and may be related to the amelioration in glucose and insulin levels.

### 4.4 Effects of acorn muffin on oxidative stress

Antioxidant activity was not statistically significant, between groups, in our study. Rakic et al. reported that the antioxidant activity of acorn extract is due to water‐soluble substances and also depends on the concentration (Rakić et al., 2006). So, our result could be associated with losing some unknown amount of antioxidant compound during acorn treatment or the amount of treated flour that was used. However, this requires further investigation into preparation methodologies to ascertain.

### 4.5 Effects of acorn muffin on anthropometric indices

In our study, no change was seen in anthropometric parameters following 8 weeks of acorn intake. It could be due to the short duration of the study and the amount of treated acorn flour that was used. In Dogan et al. study (Dogan et al., 2015), rats' weight did not significantly change after treating with Quercus extract, while the results of Shaheen et al. study (Shaheen et al., 2017) demonstrated an increase in rats' weight. However, the Quercus species examined in these studies and the amounts were different. Moreover, the aforementioned studies utilized animal models, which hinders identification of a plausible mechanism.

### 4.6 Effects of acorn muffin on appetite

Our results showed a statistically significant reduction in hunger, prospective to eat, and desire to eat, also in addition to a statistically significant increment in the feeling of fullness and satiety. These effects could plausibly relate to the presence of tannins in the acorn flour. Our result is consistent with the previous animal studies, where two investigations demonstrated a statistically significant reduction in appetite and feed intake and weight loss of fish that received tannins‐contained diet (Gaber, 2006; Omnes et al., 2017). Silanikove et al. reported goats that fed with leaves, which contain high tannins, were confronted by decreased intake and weight loss (Silanikove et al., 1996). Based on empirical studies, tannins could affect appetite in the short and long term, from twenty minutes to several weeks (Silanikove et al., 2001). Tannins may elicit a decrease in the digestion of proteins and some by‐products like volatile fatty acids, and also impair carbohydrate digestion by inhibiting α‐amylase and α‐glucosidase, hence influencing the appetite and decreasing total energy uptake (Barrett et al., 2018; Makkar et al., 1995; Robbins et al., 1987). Since impaired glucose regulation could stimulate hunger, and consequently increase food intake, the effect of acorn in decreasing appetite is considerable (Ludwig, 2002).

The current study has some limitations that should be noted. As a precautionary measure against constipation, only a small amount of acorn flour, which is commonly used in traditional society, was used. The outbreak of COVID‐19 pandemic led to difficulties in patients' access to the laboratory and caused loss to follow‐up in numerous subjects. However, this was clearly out of the operational control of the study. A final limitation was the relatively short duration of the study. Indeed, we, therefore, assert that longer duration studies are warranted.

## 5 CONCLUSIONS

The results of the current investigation indicated that daily consumption of acorn muffin (10 g acorn flour) may ameliorate HbA1c, HDL, TG, and impose decrements in appetite. More studies, with higher sample sizes and longer follow‐up, are needed to confirm acorn flour's potential beneficial health effects.

## FUNDING INFORMATION

This article was financially supported by Shiraz University of Medical Sciences, Shiraz, Iran (no. 98–01–84‐19893). However, Shiraz University of Medical Sciences had no role in the design, analysis, or writing of this article.

## CONFLICT OF INTEREST

None.

## Data Availability

The data that support the findings of this study are available from the corresponding author, upon reasonable request.
